# Coevolution in Action: Disruptive Selection on Egg Colour in an Avian Brood Parasite and Its Host

**DOI:** 10.1371/journal.pone.0010816

**Published:** 2010-05-26

**Authors:** Canchao Yang, Wei Liang, Yan Cai, Suhua Shi, Fugo Takasu, Anders P. Møller, Anton Antonov, Frode Fossøy, Arne Moksnes, Eivin Røskaft, Bård G. Stokke

**Affiliations:** 1 School of Life Sciences, Sun Yat-sen University, Guangzhou, People's Republic of China; 2 College of Life Sciences, Hainan Normal University, Haikou, People's Republic of China; 3 Department of Information and Computer Sciences, Nara Women's University, Kita-Uoya Nishimachi, Nara, Japan; 4 Laboratoire d'Ecologie, Systématique et Evolution, Université Paris-Sud, Orsay, France; 5 Department of Biology, Norwegian University of Science and Technology (NTNU), Trondheim, Norway; 6 Centre for Advanced Study (CAS), Oslo, Norway; Smithsonian Institution National Zoological Park, United States of America

## Abstract

**Background:**

Trait polymorphism can evolve as a consequence of frequency-dependent selection. Coevolutionary interactions between hosts and parasites may lead to selection on both to evolve extreme phenotypes deviating from the norm, through disruptive selection.

**Methodology/Principal finding:**

Here, we show through detailed field studies and experimental procedures that the ashy-throated parrotbill (*Paradoxornis alphonsianus*) and its avian brood parasite, the common cuckoo (*Cuculus canorus*), have both evolved egg polymorphism manifested in discrete immaculate white, pale blue, and blue egg phenotypes within a single population. In this host-parasite system the most common egg colours were white and blue, with no significant difference in parasitism rates between hosts laying eggs of either colour. Furthermore, selection on parasites for countering the evolution of host egg types appears to be strong, since ashy-throated parrotbills have evolved rejection abilities for even partially mimetic eggs.

**Conclusions/Significance:**

The parrotbill-cuckoo system constitutes a clear outcome of disruptive selection on both host and parasite egg phenotypes driven by coevolution, due to the cost of parasitism in the host and by host defences in the parasite. The present study is to our knowledge the first to report the influence of disruptive selection on evolution of discrete phenotypes in both parasite and host traits in an avian brood parasitism system.

## Introduction

Polymorphism in natural populations can evolve and be maintained as a consequence of frequency-dependent predation [1], [2], with the textbook example being industrial melanism in the peppered moth (*Biston betularia*) [1], [3]. Such polymorphism in prey populations can also result in the evolution of polymorphism in predator populations, if predators with different colours enjoy a frequency-dependent advantage during predation [4], [5]. Theoretically, other interspecific interactions acting in a frequency-dependent manner should likewise be able to produce polymorphisms in the interacting parties. Here we describe such an example of egg colour polymorphism in a brood parasite and its host. Egg colour in birds is usually a continuous character, with rare cases of discrete polymorphism in brood parasites and their hosts [6]. However, the origin of such egg colour polymorphism remains largely unknown. Here we show that a passerine host of the common cuckoo (*Cuculus canorus*) (hereafter cuckoo) has evolved three discrete egg colour morphs, but also that its brood parasite has evolved three discrete egg morphs that perfectly match those of its host.

Coevolution is defined as specialized relationships between species that lead to reciprocal evolutionary change, driven by natural selection [7]. A particularly suitable model system for studying coevolution is that of avian obligate brood parasites and their hosts [8]. These parasites lay eggs in the nests of other species (hosts), which rear the parasite offspring as their own, suffering significant fitness costs. Hosts of brood parasites frequently experience severe costs related to egg loss, misdirected parental care, and overcrowding [9]. These costs are essential as driving forces behind coevolutionary arms races between parasites and their hosts [10]. Parasitism rates, coupled with present and future fitness costs of hosts, should determine the selection pressures acting on hosts to evolve anti-parasite adaptations, and these in turn should directly influence the evolution of counter-adaptations in their associated parasites [11], [12].

Avian brood parasitism sets a unique stage for investigating microevolution, as egg colouration is the main trait under selection in both parasites and hosts. For the host, it is essential to discriminate between its own eggs and those of the parasite to prevent the loss of own offspring. For the parasite, it is crucial to mimic host eggs in order to prevent the host from rejecting its egg. A particularly well studied avian brood parasite is the cuckoo, which is widely distributed throughout the Western Palearctic and Asia, and mainly utilizes small passerine hosts. The great diversity of cuckoo egg appearance, and the many cases of striking egg mimicry with some of its host species is the basis for the “gentes theory” [10], [13]–[15]. Cuckoo gentes are tribes of females, each specializing on one or a few related host species and laying eggs of a constant type, often mimicking host eggs. Based on egg appearance, at least 17 distinct cuckoo gentes have been described in Europe [14], [16]–[18]. However, variation in egg appearance between different females in a single population is large in several host species [19], [20], making it difficult for the parasite to mimic the range of eggs present. Theoretically, such high interclutch variation in host egg appearance may lead to frequency-dependent selection among cuckoos for utilizing the most common host egg phenotype [21,22, Vikan JR, Fossøy F, Huhta E, Moksnes A, Røskaft E, Stokke BG, unpubl. data]. Alternatively, in host species having evolved exquisite egg recognition abilities coupled with disruptive selection for discrete egg morphs [23]–[26], parasites may evolve the same degree of egg polymorphism and show further within-host specialization. This could lead to further specialization where both hosts and parasites have evolved several clearly defined egg types occurring in the same geographical area. Although theoretical models suggest that discrete egg morphs can evolutionarily coexist both in host and parasite populations [27], [28], such highly advanced coevolutionary outcomes have, to our knowledge, never been documented in avian brood parasite-host systems. Clearly, there is potential for divergent evolution in both host and parasite traits; from previous studies we know that there may be marked spatial variation in coevolved adaptations among hosts and parasites in general [29] and among brood parasites and their hosts in particular [30]–[32].

In this study, we investigated whether disruptive selection may be affecting egg characteristics in ashy-throated parrotbills (*Paradoxornis alphonsianus*) and their parasite (cuckoo) in south-western China. Vinous-throated parrotbills (*Paradoxornis webbianus*), which are closely related to ashy-throated parrotbills, are known as cuckoo hosts and also displays a high degree of egg polymorphism [33], [34]. Furthermore, among the 19 species in the *Paradoxornis* genus there is apparently a pronounced variation in egg colouration with ground colours being described as white, green-white, grey, yellow, brown, reddish, pale blue and blue. In several species, eggs also contain markings and spots of various colours [35]. Given this pronounced potential for egg polymorphism, the ashy-throated parrotbill-cuckoo system should be well suited for investigations quantifying the strength of reciprocal disruptive selection leading to evolution of discrete egg phenotypes. We describe extreme egg polymorphism in the host species paralleled by corresponding egg polymorphism in the parasite. Furthermore, we experimentally test egg discrimination abilities in parrotbills to assess host defence mechanisms, and thereby selection on cuckoos for evolving mimetic eggs. Based on these analyses, we discuss the possible reasons for the observed egg polymorphism in both hosts and parasites.

## Methods

### Study area and study species

The study was performed in the Kuankuoshui Nature Reserve, Guizhou province, south-western China (28°10′N, 107°10′E) during April to July 1999–2009. The study site is situated in a subtropical moist broadleaf and mixed forest, interspersed with abandoned tea plantations, shrubby areas, and open fields used as cattle pastures.

The ashy-throated parrotbill (hereafter parrotbill) is a small passerine distributed in south-western China and northern Vietnam [36]. In our study area, the parrotbill is one of the most common bird species, breeding at forest edges and semi-open habitats, often building its nest just above the ground in dense grass or shrub (own pers. obs.).

Nests were found by systematically searching all typical and potential nest sites and by monitoring the activities of adults throughout the breeding season. We recorded date of the first egg laid, egg colour (as described below), egg and clutch size, and occurrence of brood parasitism. When a nest was found during the incubation period, eggs were floated to estimate laying date [37]. Nest predation rates were calculated for three years of the study by including nests that were used to estimate occurrence of brood parasitism.

In the study area, several cuckoo species co-occur, of which three belong to the *Cuculus* genus. This situation poses a potential risk that ashy-throated parrotbills are utilized by more than one parasite species. However, molecular analyses have confirmed that chicks hatching from white, pale blue and blue parasite eggs in ashy-throated parrotbills in the study area belong to only one species, the common cuckoo [Yang C, Wei L, unpubl. data].

### Quantification of egg colour and size

During the course of the study we discovered that parrotbills laid immaculate eggs which could be classified based on human vision in three discrete morphs: white, pale blue and blue eggs. However, avian and human visual systems differ in several respects. For instance, many bird species have ultraviolet-sensitive (UVS) photoreceptors as well as oil droplets that are absent in the human eye [38], [39]. Therefore, we obtained spectral reflectance from 33 host clutches in 2009 allowing us to describe egg colour objectively and explore the degree of egg morph differentiation in relation to the avian visual system. We measured one randomly selected egg per clutch and summarized its reflectance as the mean of six measurements per egg (two at the blunt, two at the middle, and two at the sharp parts of the egg). To account for the differential stimulation of the four avian cone types, we mapped the spectra onto Goldsmith's [40] tetrahedral colour space that has recently been recommended for analyses of colour patterns as processed by tetrachromatic visual systems [41]. We used the average spectral sensitivity curves for UVS-type retinas provided by Endler & Mielke [42]. Essentially, each spectrum is represented by a point in a tetrahedron, in which the vertices correspond to exclusive stimulation of the ultraviolet (UV), blue (B), green (G) and red (R) -sensitive cones, respectively, in the avian eye. Each colour point can be described by its spherical coordinates (θ, φ, r), where angles θ and φ represent the horizontal (RGB) and vertical (UV) components of hue, respectively, whereas r is the length of the colour vector in chroma or colour saturation (for more details see [41]). To visualize hue distributions independently of chroma, we mapped colours onto a unit sphere centred on the achromatic origin by using the Robinson projection, where θ [−π; π] corresponds to longitude, and φ [−π/2; π/2] to latitude [43]. As a measure of achromatic brightness, we calculated normalized brilliance following Stoddard & Prum [41]. Because only two pale blue clutches were available, we restricted our statistical comparisons of chroma and brilliance to the white and deep blue host egg types.

Egg size was measured by using a digital caliper. Mean size of host eggs for each clutch was used in calculations of volume according to the formula by Hoyt [44].

### Egg rejection experiments

In order to investigate fine-tuned egg recognition abilities in the host, we experimentally parasitized parrotbill nests (during 2007–2009) using seven different types of eggs which could be classified into a continuous range of contrasts between host and parasite eggs: (1) conspecific blue egg (conspecific, blue); (2) conspecific white egg (conspecific, white); (3) model parrotbill-sized pale blue egg (model conspecific, pale); (4) model parrotbill-sized egg that were intermediate in colour between white and pale blue (model conspecific, white-pale); (5) model parrotbill-sized egg that were intermediate in colour between pale blue and blue (model conspecific, pale-blue); (6) model cuckoo-sized blue egg (model cuckoo, blue); and (7) model cuckoo-sized white egg (model cuckoo, white). The contrast between parasite and host eggs was scored on a scale from 1 (perfect mimicry) to 5 (non-mimetic) (Table 1, see also [14]). The model eggs used in groups (3–7) were made of plaster and painted so as to mimic host eggs to varying degrees, while real parrotbill eggs (1–2) were left unpainted and represented either very low or very high contrast compared to host eggs (Table 1). Model conspecific eggs (3–5) were not significantly different in volume from real parrotbill eggs (F = 0.36, df = 1, 82, P = 0.55). Model eggs used in experimental groups (6) and (7) were similar in colour to eggs in experimental groups (1) and (2), but were made according to typical egg sizes of cuckoo eggs. The cuckoo-sized model eggs were not significantly different in volume compared to natural cuckoo eggs found in parrotbill nests (F = 0.05, df = 1, 35, P = 0.82).

**Table 1:** Results from experimental parasitism of ashy-throated parrotbill clutches.

			Rejection behaviour				
Host	Parasite	Contrast	Deserted	Ejected	Accepted		Total
*Conspecific eggs*							
blue	blue	1	1	0	12	(92.3)	13
white	white	1	2	0	18	(90.0)	20
blue	white	5	1	10	1	(8.3)	12
white	blue	5	2	15	2	(10.5)	19
*Model eggs*							
blue	cuckoo, blue	1	0	3	10	(76.9)	13
white	cuckoo, white	1	0	6	10	(62.5)	16
blue	conspecific, pale-blue	2	0	6	6	(50.0)	12
white	conspecific, white-pale	2	0	9	3	(25.0)	12
blue	conspecific, pale	3	0	9	3	(25.0)	12
white	conspecific, pale	3	0	10	2	(16.7)	12
blue	conspecific, white-pale	4	0	10	2	(16.7)	12
white	conspecific, pale-blue	4	0	11	1	(8.3)	12
blue	cuckoo, white	5	0	12	1	(7.7)	13
white	cuckoo, blue	5	0	18	1	(5.3)	19

Contrast  =  contrast between host and parasite eggs on a scale from 1 (low) to 5 (high). White  =  white egg, white-pale  =  egg with intermediate colour between white and pale blue, pale  =  pale blue egg, pale-blue  =  egg with intermediate colour between pale blue and blue, blue  =  blue egg. Three types of eggs were used; natural conspecific eggs, model cuckoo-sized eggs (cuckoo) and model parrotbill-sized eggs (conspecific). Numbers in brackets are % acceptance within each combination.

Use of model eggs in rejection experiments has been questioned in previous studies [45]. In order to examine the influence of using model eggs in studying rejection behaviour, we compared rejection of natural cuckoo eggs and model cuckoo-sized eggs (thus controlling for size) within both low (contrast  = 1) and high (contrast  = 5) contrast groups (i.e. contrast between parasite and host eggs). In both natural and model cuckoo eggs (6, 7) low contrast eggs were rejected significantly less than high contrast eggs (natural parasitism: 2/14 vs. 4/4, experimental parasitism: 9/29 vs. 30/32, Fisher's exact tests, P = 0.0049 and P<0.0001, respectively). Furthermore, within both low and high contrast groups, there were no significant differences in rejection of natural versus model cuckoo eggs (low: 2/14 vs. 9/29, high: 4/4 vs. 30/32, Fisher's exact tests, P = 0.29 and P = 1.00, respectively). Therefore, results from model and real egg experiments were merged in the analyses of fine-tuned egg rejection behaviour. Some previous studies have also found an effect of egg size on rejection behaviour [46]–[48]. We investigated the possible influence of egg size on rejection by comparing rejection of real conspecific eggs (1, 2) and natural cuckoo eggs in relation to contrast between parasite and host eggs. Within both low (contrast  = 1) and high (contrast  = 5) contrast groups, there were no significant differences in rejection of real conspecific eggs and natural cuckoo eggs (low: 3/33 vs. 2/14, high: 28/31 vs. 4/4, Fisher's exact tests, P = 0.63 and P = 1.00, respectively). Therefore, we also merged results from experiments using eggs of different size in the analyses of fine-tuned egg rejection behaviour.

In all egg experiments, one host egg was exchanged with one experimental egg. All experiments were carried out on the day after clutch completion or at the beginning of incubation. Nests were monitored on a daily basis for six days after experimental parasitism in order to record the response, which was classified as acceptance (foreign egg(s) warm and being incubated) or rejection (foreign egg(s) gone or left cold in the nest).

Data analyses were performed in SPSS 17.0 for Windows.

## Results

### Nest predation, natural parasitism and egg characteristics

**Figure 1:**
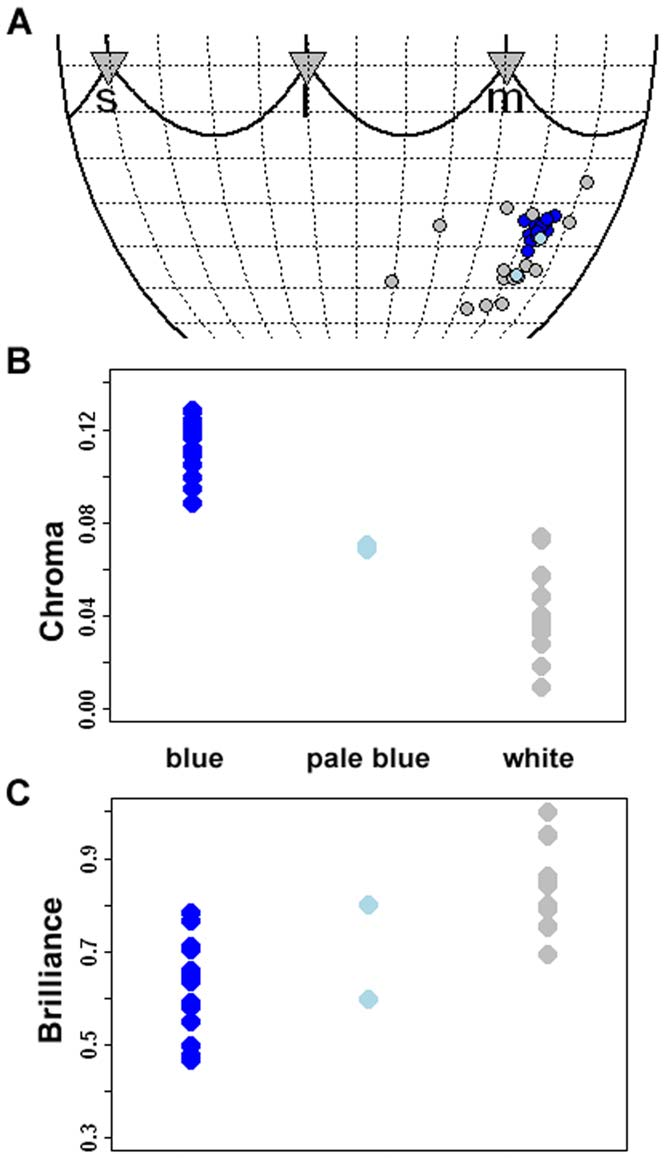
Aspects of colour and brightness of ashy-throated parrotbills eggs. Blue, light blue and grey points indicate blue (16 eggs), pale blue (3 eggs) and white (15 eggs) egg morphs. (A) Robinson projection of egg colour hues. Grey triangles indicate projections of the short (s), medium (m) and long (l) wavelength vertices of the tetrahedron. For illustration clarity, only the bottom part of the sphere is shown, and hence the projection of the ultraviolet (uv) wavelength projection is omitted. (B) Chroma or colour saturation. (C) Normalized brilliance as a measure of achromatic brightness. See Results for more detailed descriptions.

Analyses of egg colour showed that blue parrotbill eggs were compactly distributed in the bluish-green region of the hue space, while white eggs had a markedly more scattered hue distribution (Figure 1a). Since blue eggs were within the range of white egg hue variation, the two egg types did not have clearly distinct hues (Figure 1a). However, blue and white eggs were completely separated in chroma and also to a large extent in brilliance (Figure 1b, 1c). White eggs were much less saturated in colour and were brighter than blue eggs (chroma: Welch t = 12.65, df = 23, 643, P<0.0001; brilliance: t = −5.64, df = 19, 597, P<0.0001). Pale blue eggs were intermediate (Figure 1), but the few data points prevent statistical comparisons. These analyses clearly indicate that results from avian visual modelling agree well with classification of host egg morphs based on human vision.

**Figure 2:**
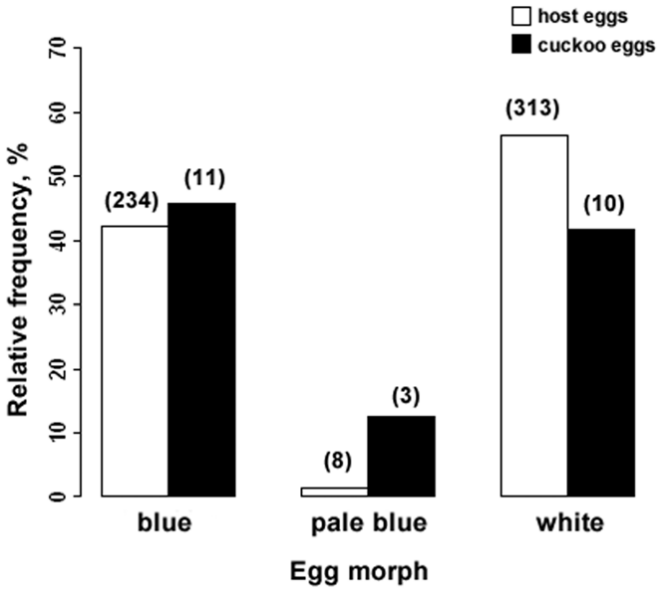
Frequency distributions of ashy-throated parrotbill and cuckoo egg morphs. Numbers above bars denote number of nests.

Within a single nest, no more than one host egg type was found, and intraclutch variation in egg appearance appeared to be very low as qualitatively assessed using human vision (no quantitative measures were made). However, host eggs were strongly dimorphic in being either white or blue, while the pale blue morph was very rare (1.4%, Figure 2). Overall, there were more white than blue host clutches (Figure 2, χ^2^ = 11.41, df = 1, P = 0.001). Interestingly, there was also a significant difference in the frequencies of clutches with white and blue eggs among years (χ^2^ = 16.04, df = 5, P = 0.007, Table 2). Only in 1999 there were more clutches with blue than white eggs, while the situation was opposite during the rest of the years. Interestingly, cuckoos also laid predominantly white and blue eggs (Figure 2), but egg morph frequencies were significantly different from those of the host (χ^2^ = 15.78, df = 2, P<0.001). This was because of relatively more pale blue eggs laid by cuckoos than their hosts, since host and parasite egg morph frequencies did not differ significantly when only the predominant white and deep blue eggs were considered (χ^2^ = 0.41, df = 1, P = 0.52).

**Table 2:** Temporal variation in distribution of egg types in ashy-throated parrotbills.

Year								
		1999	2004	2005	2007	2008	2009	Total
Egg type	white	30	52	34	88	49	60	313
	pale	0	0	2	1	2	3	8
	blue	42	18	25	64	34	51	234
	Total	72	70	61	153	85	114	555

Nest predation rates were quite substantial, but with no significant difference between nests containing white or blue host eggs (Table 3). The overall rate of cuckoo parasitism in parrotbills was 4.3% (N = 555 nests), with no significant differences between years (Table 4, χ^2^ = 7.14, df = 5, P = 0.21). No cases of multiple parasitism were found. A cuckoo egg was significantly more likely to be found in a host nest of the corresponding egg morph, rather than in a ‘wrong’ one (19/24 vs. 5/24; χ^2^ = 8.17, df = 1, P = 0.004). Furthermore, parasitism rate of host clutches containing pale blue eggs (25%, N = 8) was significantly higher than in clutches containing white eggs (3.83%, N = 313), and marginally higher than in clutches containing blue eggs (4.27%, N = 234; Fisher's exact tests, P = 0.043 and P = 0.054, respectively). There was no significant difference in parasitism rate between clutches containing white or blue eggs (Fisher's exact tests, P = 0.83). Overall, 33% (6/18) of the cuckoo eggs were rejected, although cuckoo eggs laid in nests with the corresponding host egg morph were significantly less likely to be rejected by the host than those laid in nests with “wrong” egg morphs (2/14 vs. 4/4; Fisher's exact test, P = 0.0049). Five out of the six rejected cuckoo eggs were ejected and one was deserted. In all cases where cuckoo eggs were accepted, the host lost all its reproductive output because the cuckoo chick ejected all host eggs upon hatching.

**Table 3:** Occurrence of nest predation in ashy-throated parrotbills.

Predation rates (%) on various egg types					
		white	blue	Total	Chi-square test
Year	1999	(53.3) 30	(42.9) 42	(47.2) 72	χ^2^ = 0.77, df = 1, P = 0.38
	2005	(26.5) 34	(36.0) 25	(30.5) 59	χ^2^ = 0.62, df = 1, P = 0.43
	2009	(48.3) 60	(49.0) 51	(48.6) 111	χ^2^ = 0.005, df = 1, P = 0.94
	Total	(43.5) 124	(44.1) 118	(43.8) 242	χ^2^ = 0.007, df = 1, P = 0.94

Predation rates are provided as % (in brackets) with total number of nests monitored. Differences in predation rate between egg types are tested with Chi-square tests.

**Table 4:** Number of ashy-throated parrotbill nests parasitized by common cuckoos.

		Year						
Host	Parasite	1999	2004	2005	2007	2008	2009	Total
Blue	white	1	0	0	0	0	0	1
Blue	pale	0	1	0	0	0	0	1
Blue	blue	3	0	1	2	2	0	8
Pale	pale	0	0	0	0	1	1	2
White	white	1	2	1	2	2	1	9
White	blue	0	2	0	0	1	0	3
All		5 (6.9)	5 (7.1)	2 (3.3)	4 (2.6)	6 (7.1)	2 (1.8)	24 (4.3)
Total		72	70	61	153	85	114	555

White  =  white egg, pale  =  pale blue egg, blue  =  blue egg. “All” refers to the total number of nests parasitized (parasitism rate (% nests parasitized) in brackets). “Total” refers to the total number of nests recorded, whether parasitized or not, and was used in the calculation of parasitism rate.

There was no significant difference in host egg volume between 2008 and 2009 (F = 0.012, df = 1, 63, P = 0.91). Furthermore, there was no significant difference in volume (F = 0.10, df = 1, 60, P = 0.75) or clutch size (F = 0.01, df = 1, 188, P = 0.91) between blue and white host clutches. Natural cuckoo eggs were approximately twice the size in volume of host eggs (mean ± SD, cuckoo: 2.63 cm^3^ (±0.18, SD), N = 6; host: 1.33 cm^3^ (±0.08, SD), N = 69, F = 1034.52, df = 1, 74, P<0.001).

### Egg recognition in parrotbills

**Figure 3:**
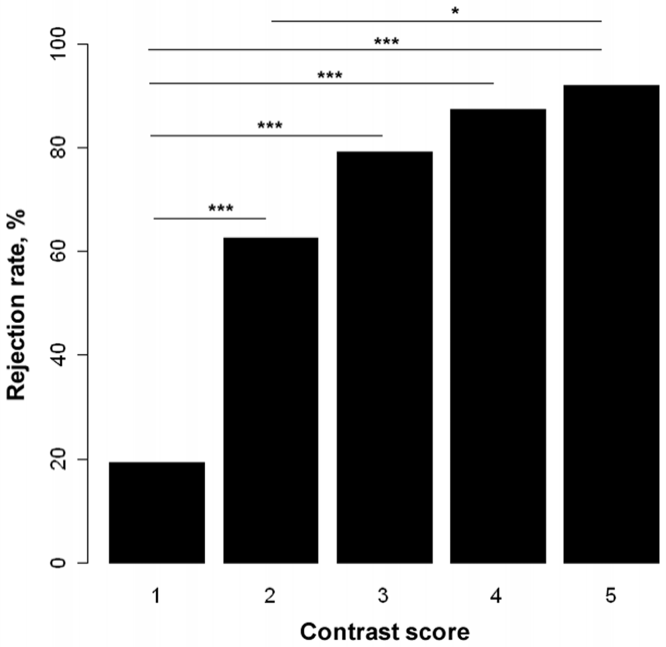
The relationship between contrast in egg appearance and egg rejection rate in ashy-throated parrotbills. 1 =  lowest and 5 =  highest contrast. Pairwise differences in rejection rates between contrast levels were tested using Fisher's exact tests. Holm's [76] sequential method was applied as a P-value adjustment procedure. *P = 0.01, ***P<0.0001.

We examined fine-tuned egg rejection behaviour of parrotbills by comparing rejection of parasite eggs differing in egg colour contrast compared to host eggs (Table 1). There was a significant effect of contrast between parasite and host eggs on probability of rejection, although there appeared to be a threshold in egg recognition abilities. When contrast was below or above a threshold value (contrast ≈2), parrotbills would either accept or reject most parasite eggs, respectively (Figure 3). There was no significant difference between time of rejection of the parasite egg after experimental parasitism and contrast between host and parasite eggs (F = 1.17, df = 4, 124, P = 0.33). Mean (± SD) day of rejection for all contrast groups combined was 1.95 days (±0.94, SD) (N = 125).

## Discussion

To our knowledge, we report the first empirical evidence for disruptive selection on egg colours in an avian brood parasite and its host. Both ashy-throated parrotbills and common cuckoos have evolved egg polymorphism within a single population. In both species, the most common egg colours were white and blue, and hosts with the two egg colours experienced similar parasitism rates. The pale blue egg type appeared to be rare in both hosts and parasites. Egg rejection experiments showed that selection on parasites for countering the evolution of host egg types is evidently strong because hosts generally have evolved good abilities for rejecting even partly mimetic eggs. Furthermore, selection on hosts for evolving discriminating abilities is strong, because successful parasitism always leads to a complete loss of reproductive output for the host. It is interesting to note that Chavigny & Le Du [49], who studied cuckoo parasitism on Moussier's redstarts (*Phoenicurus moussieri*) in North Africa, also found white and blue host and cuckoo eggs. However, they collected data on a larger spatial scale potentially involving multiple host and parasite populations. In addition, there are no reports on host rejection abilities in Moussier's redstarts, making it impossible to compare that particular system with the cuckoo-parrotbill system.

Parrotbill eggs were polymorphic with an uneven distribution of egg morphs. The vast majority of clutches were clearly dimorphic, consisting of either pure white or blue eggs and only a very small proportion of nests contained intermediate pale blue eggs. Interestingly, there were overall more clutches containing white than blue eggs, which is opposite to vinous-throated parrotbills in Korea where blue eggs were more common than white ones, although with some temporal and spatial variation [33]. As shown by avian visual modelling, the two main egg morphs in the parrotbill were indeed discrete with respect to colour saturation and brightness, justifying the classification by the human eye (see also [50]). Remarkably, all three egg types were also found in the cuckoo, where there were predominantly white and blue eggs with few pale blue ones, nicely mirroring the situation in parrotbill hosts. Pale blue eggs were more frequent among cuckoos than parrotbills, but the biological significance of this difference is unclear, as it may simply have resulted from the huge inequality in sample sizes of host and cuckoo eggs. As evident from both natural and experimental parasitism, parrotbills showed highly developed egg discrimination abilities, rejecting almost all foreign eggs of the “wrong” morph, but accepting the corresponding one. These findings are consistent with the scenario that the present state of egg phenotype co-adaptation is an outcome of strong disruptive selection on both host and parasite maintaining a high interclutch variation in egg appearance within the population. Support for the hypothesis that brood parasites may actually be responsible for high variation in host egg phenotypes has previously been found [20], [23], [51].

A simple but possible evolutionary scenario for the occurrence of highly divergent host egg phenotypes is suggested by the presence of pale blue eggs in the study population. It is surprising that this intermediate egg type, which was very rare in the host, was also present in the cuckoo. One possible explanation is that the pale blue egg may be a vanishing egg morph that was once optimal. The pale blue egg may have been the only or the most common host egg type at earlier stages in the coevolutionary arms race with cuckoos. Thus, the evolution of perfect egg mimicry by the cuckoo, resulting in an immaculate pale blue egg type with little variation is likely to have occurred relatively rapidly, giving a huge fitness advantage to the cuckoo. The resulting fitness costs for the host due to parasitism have likely imposed strong selective pressure on the host for evolution of extreme phenotypes such as white and blue eggs, thereby providing an opportunity to recognize pale blue cuckoo eggs. In turn, the change in appearance of host eggs coupled with high rejection rates of non-mimetic eggs should lead to strong disruptive selection in cuckoos for matching either of the two extreme host egg phenotypes. Therefore, the state of affairs seen today in this particular host-parasite system is that cuckoos have “caught up” with the host in term of egg matching, but the egg polymorphism present in the host should favour active host selection by individual cuckoo females that lay in nests with the corresponding host egg type to avoid egg rejection.

Alternatively, the ancestral state could be either white or blue host eggs. Interestingly, only blue cuckoo eggs have been found in vinous-throated parrotbills in Korea, although the host lays both blue and white eggs [34]. However, in Taiwan and eastern Manchuria, only the blue egg morph has been found in the vinous-throated parrotbill [35]. Cuckoos are absent at least in Taiwan [52], hence, blue host eggs could have been the ancestral type in Korea and China, but hypothetically cuckoo parasitism may have resulted in evolution of polymorphic host eggs in these populations. Furthermore, the remaining six species of *Paradoxornis* parrotbills that are most closely related to ashy-throated and vinous-throated parrotbills all lay pale blue, sky blue or deep blue eggs [35]. Thus, apparently white eggs only appear in those two species of *Paradoxornis* parrotbills that are known to be utilized by cuckoos. This knowledge strengthens the notion that disruptive selection acts to produce extreme egg phenotypes in parasitized parrotbill populations, with pale blue or blue host and cuckoo eggs being the ancestral state. The parrotbill system seems to be one step ahead in the co-evolutionary arms race than a similar system in Fennoscandia. In the brambling (*Fringilla montifringilla*), a species used as host by the cuckoo, there is also very high interclutch variation in appearance of host eggs [53], although the colour phenotype of host eggs are continuous, and the cuckoo has evolved mimicry only for the intermediate section of this range rather than the extremes (Vikan JR, Fossøy F, Huhta E, Moksnes A, Røskaft E, Stokke BG, unpubl. data).

Most cuckoo eggs in our study population were found in nests with the corresponding host egg type and were accepted while the few eggs found in the “wrong” nests were all rejected. This finding suggests that cuckoos may have evolved a strategy of selecting nests with the corresponding egg type. Such active host selection would enable cuckoos to persist using a strongly rejecting host that lays dimorphic eggs. There is suggestive evidence for this idea even in cuckoo-host systems with continuously distributed egg phenotypes [54], [55]. Although cuckoos have evolved the same degree of egg polymorphism as their parrotbill hosts, our study cannot provide conclusive evidence for this hypothesis. Precisely because parrotbills are such good rejecters of poorly matching eggs, we may have failed to detect some cuckoo eggs laid in the ‘wrong’ nest, implying potential bias towards good matches. Future studies using radio-telemetry would allow detailed investigations of host selection by parrotbill cuckoos.

Parasitism rates on parrotbills in our study area were consistent in time and comparable to long term rates in several major host species in Europe [56]–[58]. Is a parasitism rate of approximately 5% as found in the present study a sufficiently strong selection pressure for evolving host defences? The answer is apparently yes, as other hosts experiencing similar parasitism rates have evolved adaptations like egg rejection [10]. Furthermore, there is obviously significant spatial [59] and temporal [60], [61] variation in parasitism rates. This indicates that the strength of selection for evolving traits important in coevolutionary interactions may vary significantly in both space and time. We do not possess data on parasitism of ashy-throated parrotbills from other areas, but closely related vinous-throated parrotbills in Korea experience comparable parasitism rates [34]. Finally, our estimated parasitism rates may be underestimated since, according to our results, parrotbills reject even partly mimetic eggs quickly and to a large extent. Therefore, it is possible that some cases of parasitism have been missed.

Nest predation is an important selective agent that should be taken into account when investigating aspects related to breeding in birds [62]–[64]. Many bird species have evolved eggs that appear more cryptic in colour against the nest background than the ancestral white egg colour [6], which may lower the probability of detection by predators [65]–[67]. Even blue eggs may appear cryptic in open nests built in dense vegetation [6,68, but see 65]. However, a comparative analysis investigating factors explaining intraclutch variation in egg appearance among European and North-American passerines obtained no evidence for higher predation rates in species with more diverse eggs within clutches [69]. Furthermore, several previous studies have shown that parental activity at nests, nest size and nest concealment rather than egg colour are the most important factors explaining variation in nest predation [65], [70]–[72]. In line with this, Kim et al. [33] found that nest failure in vinous-throated parrotbills was related to nest height, which is indicative of nest concealment, rather than egg colour. The results from the present study revealed a considerable nest predation rate, but also that the likelihood of predation was not significantly different between nests with white and blue eggs. Therefore, it is reasonable to conclude that nest predation is not responsible for the evolution of egg polymorphism in ashy-throated parrotbills.

The final outcome of the interactions between cuckoos and parrotbills in terms of egg phenotypes is obviously difficult to predict based on the results from the present 11-year study. In theory however, according to the Red Queen hypothesis, cuckoos may actually be able to drive oscillations in parrotbill genotype frequencies through negative frequency-dependent selection [73]–[75]. Therefore, if we reasonably assume that there is a genetic basis behind egg phenotypic expression, cuckoos may be responsible for temporal variation in frequencies that would ensure maintenance of a long-term stable polymorphism of parrotbill egg phenotypes. Our results indicate that there is actually temporal variation in frequencies of host egg phenotypes (more clutches with blue than white eggs in 1999, but the opposite during 2004–2009). Therefore, it will be very interesting to monitor changes in both parrotbill and cuckoo egg phenotype frequencies in the years to come.

In conclusion, the existence of polymorphic host and cuckoo eggs is likely to have evolved through coevolutionary interactions favouring more extreme egg phenotypes. Due to the high costs of parasitism, there should be strong disruptive selection on host egg phenotypes followed by selection on cuckoos due to high rejection rates of non-mimetic eggs by the parrotbills. Therefore, cuckoos should select hosts that produce eggs of their corresponding type. Thus, our results indicate disruptive selection on both host and parasite egg phenotypes driven by the cost of parasitism (host) and by host defences (parasite).
